# Exploring young women’s reasons for adopting intrauterine or oral emergency contraception in the United States: a qualitative study

**DOI:** 10.1186/s12905-020-0886-z

**Published:** 2020-01-28

**Authors:** Shelly Kaller, Aisha Mays, Lori Freedman, Cynthia C. Harper, M. Antonia Biggs

**Affiliations:** 1 Advancing New Standards in Reproductive Health (ANSIRH), Bixby Center for Global Reproductive Health, Department of Obstetrics, Gynecology & Reproductive Sciences, University of California, San Francisco, 1330 Broadway, Suite 1100, Oakland, CA 94612 USA; 2 Bixby Center for Global Reproductive Health, Department of Obstetrics, Gynecology & Reproductive Sciences, University of California, San Francisco, 3333 California St, Suite 335, San Francisco, CA 94143 USA

**Keywords:** Intrauterine device, IUD, Emergency contraception, Long-acting reversible contraception, women’s health, qualitative research

## Abstract

**Background:**

The recent focus on increasing access to long-acting reversible contraceptive methods has often overlooked the diverse reasons why women may choose less effective methods even when significant access barriers have been removed. While the copper intrauterine device (IUD) is considered an acceptable alternative to emergency contraception pills (ECPs), it is unclear to what extent low rates of provision and use are due to patient preferences versus structural access barriers. This study explores factors that influence patients’ choice between ECPs and the copper IUD as EC, including prior experiences with contraception and attitudes toward EC methods, in settings where both options are available at no cost.

**Methods:**

We telephone-interviewed 17 patients seeking EC from three San Francisco Bay Area youth-serving clinics that offered the IUD as EC and ECPs as standard practice, regarding their experiences choosing an EC method. We thematically coded all interview transcripts, then summarized the themes related to reasons for choosing ECPs or the IUD as EC.

**Results:**

Ten participants left their EC visit with ECPs and seven with the IUD as EC option. Women chose ECPs because they were familiar and easily accessible. Reasons for not adopting the copper IUD included having had prior negative experiences with the IUD, concerns about its side effects and the placement procedure, and lack of awareness about the copper IUD. Women who chose the IUD as EC did so primarily because of its long-term efficacy, invisibility, lack of hormones, longer window of post-coital utility, and a desire to not rely on ECPs. Women who chose the IUD as EC had not had prior negative experiences with the IUD, had already been interested in the IUD, and were ready and able to have it placed that day.

**Conclusions:**

This study highlights that women have varied and well-considered reasons for choosing each EC method. Both ECPs and the copper IUD are important and acceptable EC options, each with their own features offering benefits to patients. Efforts to destigmatize repeated use of ECPs and validate women’s choice of either EC method are needed to support women in their EC method decision-making.

## Background

While the copper intrauterine device (IUD) is considered an acceptable alternative to emergency contraception pills (ECPs) [1, 2], it is unclear to what extent low rates of provision and use are due to patient preferences versus structural access barriers. The copper intrauterine device (IUD) can be used as emergency contraception (EC), provides ongoing contraception for up to 12 years, and is more effective than oral EC pills (ECPs), particularly if used 5 days or more after unprotected sex or shortly before a woman ovulates [1–5].

When compared to ECPs, few providers offer the IUD as EC, and awareness of this option among providers and patients is low [6–9]. In a survey of multispecialty providers who serve reproductive-aged patients, the majority (84%) knew that the IUD could be used as EC, but only 36% provided it, as compared to 81% who provided oral levonorgestrel ECPs, the most common type of ECPs [2, 6]. While very few women (< 10%) have heard of the IUD as EC [9], when asked about it, they report interest in its long-term, highly effective nature and ease of use, while also expressing concerns about placement and negative side effects [9–13].

Limited availability of the IUD as EC, along with its high cost, present significant barriers to women interested in this option, barriers that are even more pronounced among younger women [14–16]. The combination of access barriers and limited provider awareness and training on the IUD as EC [17–20], makes it difficult to discern to what extent patient preferences contribute to the low rates of IUD as EC use and provision.

The recent focus on increasing access to long-acting reversible contraceptive methods has often overlooked the diverse reasons why women may choose less effective methods, even when significant access barriers have been removed. Much work in the past decade has focused on decreasing structural barriers by increasing availability of and provider training on the IUD [21], in the hopes that the method’s high efficacy could provide a solution to preventing unplanned pregnancy. However, in order to provide high quality, non-coercive contraceptive care, providers need to ensure that their enthusiasm for a particular contraceptive method like the IUD is matched by patients’ interest. While we have existing research on young women’s attitudes towards IUDs as a general method [22–24], their experiences and perspectives regarding their choice between the IUD as EC and ECPs, is largely missing from research. Interviews with women presenting for EC at family planning clinics in Utah identified cost and fear of side effects as significant factors in choosing ECPs over the IUD as EC [11]. Building on this evidence, this study seeks to gain a deeper understanding of women’s perspectives on their EC options in clinical settings where access to the IUD as EC and oral ECPs, including ulipristal acetate and levonorgestrel, is standard practice, IUD-trained providers are available, and all FDA-approved methods are available at no cost, through the state’s family planning program. By interviewing young women in settings that have overcome the predominant structural barriers to IUD and ECP access, this study was able to explore more deeply the other factors that may influence method uptake, including prior experiences with contraception and attitudes toward EC methods.

## Methods

### Participant recruitment

Using a purposive sampling approach, we aimed to recruit approximately 20 women accessing EC services, evenly distributed between those choosing ECPs and the IUD as EC, a number we estimated to be feasible and sufficient to explore our research question. From September 2015 to January 2016, we invited patients from three San Francisco Bay Area youth-friendly clinics to participate in semi-structured telephone interviews regarding their experiences choosing an EC method. We selected these clinics because they offered ECPs and the IUD as EC as standard practice, in order to minimize access as a deciding factor. Women ages 30 and under were eligible to participate. We selected this age group because the vast majority of ECP users are within this age range and they may present distinctive counseling preferences and needs given their unique developmental stage and barriers they face accessing care [25, 26].

We recruited patients by posting and distributing flyers in waiting rooms and during the EC counseling visit. After clinic staff counseled the patient on the efficacy and side effects of the EC method/s available and the patient decided on an EC method, a clinic-based study recruiter shared a study description with eligible patients, then obtained verbal consent, contact information, and scheduling preferences from interested patients for the interviewers to contact them. Patients were also able to contact the research team directly after having seen a study flyer. In those cases, the interviewer verified eligibility and obtained verbal consent before conducting the interview. The informed consent process included an overview of the research team and study focus, explaining that the purpose of the study was to learn about women’s experiences making choices about the kinds of birth control methods they use and when. Within 2 days of receiving patients’ contact information and scheduling preferences, one of three female interviewers trained in in-depth interviewing, and one of whom was bilingual in English and Spanish, contacted the participant to schedule the telephone interview. Participants’ first names and contact information were stored separately from their interview responses in a password-protected file, linked by a unique identifier. The three interviewers are co-authors and have advanced degrees in medicine, public health, and psychology, and extensive experience in reproductive health. One interviewer identifies as non-Hispanic white, the other as Hispanic white, and the third as African American.

### Data collection

We designed the interview guide to be non-leading and to prompt participants to share their thoughts and experiences. To contribute to development of the guide, we conducted three pilot interviews which we did not include in the analysis. The final guide included both closed-ended and open-ended questions. Closed-ended questions confirmed eligibility and asked contraceptive method received, whether the patient had used ECPs or the IUD before, and demographics. Domains of the open-ended questions included prior experiences with contraception, awareness of and attitudes toward EC and other contraceptive methods, and visit experience, including counseling. Interviewers received training on the interview guide, how to use neutral, non-leading probes, and how to be non-judgmental and affirmative in order to be supportive of interviewees and elicit honest responses. We interviewed participants in their preferred language (English or Spanish) within 1 month of their EC visit. Interviewers obtained participants’ verbal consent to audio-record the interview. The interviews were conducted by telephone, in a location of the participant’s choice, and lasted approximately 30 min. The interviewer began the interview by confirming the participant was in a private place where she felt comfortable talking about her experiences with contraception. We did not confirm whether anyone else was present at the participant’s location. The interviews did not always follow the same order as the interview guide, but covered the same domains. After the interviews, the interviewer summarized each interview in a short analytic memo to record initial reflections. Participants received a $25 gift card as a token of appreciation for participating in the interview. The research team did not contact participants again after completion of the interview and participants’ receipt of the gift card. University of California San Francisco's Institutional Review Board approved all study materials and procedures.

### Qualitative analysis

The analytic team comprised the three previously described interviewers. During the data collection phase, the team met weekly to debrief, reflect on the interviews, and examine how our role as researchers may affect the conduct and interpretation of the interviews. We took an iterative, grounded approach to data collection in that we allowed ongoing insights to deepen our understanding of respondents’ experiences and to improve the interview process in real time. Once we had completed data collection, the analytic team conducted thematic analysis with an interpretive constructivist lens which elevates respondents’ understandings of their preferences and needs over external indicators [27]. We used Dedoose data management web application to code interview transcripts. After reviewing an initial set of interviews, two study authors independently generated a list of codes, including both a priori codes developed from the research topics and emergent codes that arose from the interviews and analytic memos. These codes were related to prior experiences with contraception, circumstances in seeking EC, and knowledge and attitudes about the methods. Both authors revised the code list iteratively after discussion and consensus and consulted a third author to discuss the code list, until a final list of codes was generated. Two of the authors then applied the final list of codes to all interviews using Dedoose, when needed applying multiple codes to the same excerpt or overlapping part of an excerpt, or super-ordinate and sub-ordinate “parent” and “child” codes to indicate levels of structure. This allowed for nuances in the coding and integration of both a priori and emergent codes. After coding the transcripts, the analytic team then systematically summarized coded excerpts related to reasons for choosing an EC method. We compared themes for participants who left the visit with ECPs versus the IUD as EC, identifying overlap and differences in how the themes applied to the two groups. The team developed thematic summaries, which we discussed and revised as a group once reaching agreement of interpretation. We present the themes below separately for women who chose ECPs and women who chose the IUD as EC.

## Results

We interviewed 17 women over the course of 5 months. Five additional patients expressed initial interest to the clinic-based study recruiter in participating in the study; two of those declined consent to participate before sharing their contact information with the research team; three others shared their contact information, but ultimately did not schedule an interview. We ceased recruitment after we noted sufficient repetition of themes that meaningfully addressed the research question. All participants were ages 16–30 and seeking ECPs for a recent sexual encounter; none were originally seeking the IUD as an EC option. The majority of participants were Latina (*n* = 11), under 20 years old (*n* = 9), had a high school education or less (*n* = 9). Most did not want to have a baby for at least 3 years (*n* = 15) with the remaining two never wanting to have a baby (Table 1). Seven participants left their visit with the IUD as EC and ten participants left with ECPs; eight of those specifically mentioned getting Plan B, a levonorgestrel ECP, while two did not specify the type. Because participants had trouble distinguishing between the different forms of ECPs received, whether ulipristal acetate or levonorgestrel, we describe these uniformly as ECPs. Women who adopted ECPs or the IUD as EC were evenly distributed by most demographic characteristics. All participants chose to be interviewed in English.

**Table 1:** Sample Description

	Total, N	Type of EC method they left visit with, N	
		ECPs	IUD
Total	17	10	7
Race/ethnicity			
Latina	11	6	5
Black	3	3	0
Asian	3	1	2
Age			
16–19	9	6	3
20–24	6	4	2
25–30	2	0	2
Highest level of education			
Some high school	5	4	1
High school grad/GED	4	2	2
College/tech degree	2	1	1
Some college	6	3	3
When participant wants a baby			
Never	2	2	0
3–7 years	6	3	3
8–12 years	9	5	4
Has ever been pregnant	4	3	1

Participants, both those who adopted ECPs or the IUD, reported visiting the clinic because they had had unprotected sex or experienced a method failure and did not want to be pregnant. All women who had an IUD placed and most who received ECPs had heard about the IUD before their visit. Few knew that the IUD could be used as EC until their current clinic visit. We were interested in learning what differentiates the women who chose ECPs versus the IUD at their EC visit, and thus examined themes related to the reasons why women left with either ECPs or the IUD.

### Reasons for choosing ECPs over the IUD

#### ECPs are a known, accessible and well-liked option

Most women were familiar with or had had positive experiences with ECPs before their visit, and would recommend them to others. Women described their appreciation for a post-coital contraceptive option that was effective and easy to obtain at their clinic. As one woman explained, “It just makes me feel a lot better knowing that I decreased the chances of being pregnant, so it just makes me happy.”

#### Repeated use of ECPs is a concern, but not a deal-breaker

Although most women held positive views about ECPs, some women who chose them also pointed to concerns that using ECPs frequently was “irresponsible”, may not always be effective, and could cause negative effects on the body, but that it was also the most responsible choice for now. While one woman chose ECPs, she also shared concerns about the method's effectiveness, “I worry if it’s actually going to work”, and its effects on future fertility, “Someone told me that you shouldn’t take too much of it [ECPs] or else it will mess you up. But I don’t know if that’s true.” Another woman described her interest in returning to the clinic later for an ongoing contraceptive method, in part because she was worried about how repeat ECP use reflected on her character:

“This is the second time I’ve ever really used it [ECPs] - I really don’t like to use Plan B [ECPs], because it makes me look like I’m irresponsible or like I don’t care. But, you know, things happen. And, if you have to do something, you got to get it done.”

Her account exposes her conflicting views about ECPs, where she values their availability, yet also is concerned that others might view her negatively for relying on ECPs. She, too, seems to dismiss her ECP use as a responsible and proactive action to prevent an unwanted pregnancy.

#### ECPs are a short-term solution, but women plan for ongoing contraception

Most women who chose ECPs planned to return to the clinic later for an ongoing method such as the injection, patch, birth control pills, or the levonorgestrel IUD, which on its own is not typically included in clinical guidelines as an EC option. Many of these women noted the benefits of the copper IUD, particularly its high and multi-year effectiveness. Some expressed interest in using it in the near future, yet they felt it was not the right choice for them at that time. One woman, who left her visit with ECPs, explained why she preferred to wait to have the copper IUD placed at a follow-up appointment:

“I think it’s better if you come back and get it done. Maybe not have it the same day… I was so nervous, because I knew I had work that day and I just - I wasn’t ready. I think it’s best to have it done [IUD placement] on a day where you’re mentally prepared for it.”

She clarified that she felt nervous about the potential pain during the IUD placement and cramping afterwards and she wanted to plan the appointment for a day when she did not have to work afterwards.

#### Disinterest in the IUD was related to negative experiences with the method, concerns about its unwanted side effects and placement procedure

For some of the women who chose ECPs, disinterest in the IUD was due to prior negative experiences using the IUD or difficulty having it placed. Three had used the IUD before and disliked it; one had a failed placement because her uterus was “too deep”. One woman chose ECPs and the contraceptive implant at her visit. She was not interested in the IUD as EC at this visit due to a previous negative experience using the IUD which included cramping, discomfort with placement, and an expulsion. When compared to the IUD, she expressed more positive attitudes about ECPs:

“When you’re stressing about being pregnant, [with ECPs] all you have to do is just swallow this little pill and hopefully your problem will go away, but with that [IUD], it just sounds like a lot. You got to get it inserted and go through that. And, also, for me personally, it’s kind of uncomfortable having someone inspect your private parts. It’s just, embarrassing.”

She felt that ECPs were a simpler and more comfortable option for her at this visit. Most of the women who chose ECPs and were not interested in the IUD received another ongoing method at the visit. A few had plans to return to the clinic for a follow-up appointment to have a method, like the implant, placed, or because they were undecided about the method they wanted to use.

When describing their reasons for not choosing the copper IUD, women frequently mentioned concerns about bleeding and cramping, in addition to discomfort with placement. For example, one woman explained how she did not like the idea of an IUD due to concerns about side effects, that it would fall out without her knowledge, and that she would forget to have it removed after it expired. “I also heard with the IUD, it kind of makes your cramping heavier and your blood a little heavier, so that’s another reason why I just said no to it.” Another woman who had a follow-up appointment to have a contraceptive implant placed also shared, “I really wouldn’t want nothing going inside of me, and especially feeling those cramps.” A different woman had used the levonorgestrel IUD for approximately 5 years after getting it placed immediately following an abortion when she was 16 years old. Her experience with the IUD was positive and she was interested in having another IUD placed. She shared her preference for the levonorgestrel IUD over the copper IUD because of its bleeding profile, “It [the copper IUD] sounds awesome besides the part that … you’re going to bleed, because I like not having my period.”

#### Lack of awareness prevents IUD as EC uptake

While familiarity of ECPs is a reason for choosing them, lack of awareness of the IUD as EC option prevents its uptake. Four women said that their provider did not tell them about the IUD as EC option at their visit and were unaware that the IUD could be used as EC until it was brought up in the interview. Three expressed varying levels of interest in adopting it, had they known about it. One woman explains that she would have considered the copper IUD had she been offered it, particularly because of its higher efficacy compared to ECPs; a second woman expressed some concerns about the IUD’s side effects and placement, yet was considering the levonorgestrel IUD as a method; a third was interested in the levonorgestrel or possibly copper IUD at her visit, but was told there was not a provider available to place one that day and was not informed that the copper IUD could be used as EC.

### Reasons for adopting IUD as EC

#### Women were familiar with and already interested in the IUD as an ongoing method

Nearly all of the women who adopted the IUD as EC (six out of seven) were already considering or planning to get the IUD before the EC visit. One other knew she wanted an effective, long-acting, and discreet method and learned about the IUD at her EC visit. None of the women had used an IUD before. They explained that they chose the copper IUD primarily because of its long-term efficacy, invisibility, lack of hormones, longer window of post-coital utility, and a desire to not rely on ECPs. One woman wanted a long-acting, non-hormonal method she could forget about and had been considering the IUD “for a while”. She shared, “It’s nice to not have to worry about any accidents, because [the IUD] does work as an emergency contraceptive too... And, I’m glad I don’t have to worry about it for 10-12 years.”

Some of the women who adopted the IUD as EC were familiar with the IUD because of friends’ and family members’ personal experiences, influencing their decisions to adopt the IUD at the EC visit. One woman described how her friends’ experiences with the IUD, combined with her own contraceptive preferences, affected her choice for the IUD. She described being “curious [about the IUD] because some of my friends told me about it before” and “love it”, but that she did not decide to use the IUD until she had discussed it with her provider.

#### Efficacy is a primary reason for choosing the IUD as EC

When comparing ECPs and the IUD as EC and considering which ongoing method to choose, the high efficacy of the IUD was a key consideration for some women. One woman was nervous about the placement process and side effects from an IUD, but ultimately chose it because of its efficacy as EC, as well as an ongoing contraceptive method. She explained that if a friend came to her because she was worried she might get pregnant after unprotected sex, she would recommend “the IUD because it’s 8 times more effective and [if] it has been some time, Plan B [ECPs] may not work for her… everyone is different. However, the IUD is effective no matter who you are.” Two women who learned about the IUD as EC option at their visit chose the copper IUD because their provider told them that ECPs may not be effective based on the timing of unprotected sex and their menstrual cycle.

#### Women chose the IUD due to worries about effects of ECPs

As with the women who chose ECPs, women who chose the IUD also voiced concerns that frequent use of ECPs might “damage something in your system”, affect one’s fertility, was irresponsible, and that ECPs do not reliably prevent pregnancy. One woman who had used ECPs regularly, now wanted an IUD. She explained, “I think we’re using it [ECPs] too much, and I don’t know if it is bad for my body or the effects of it.” Similarly, a woman who was interested in a consistent, reliable, and non-hormonal method said:

“This is the third time in my life that I’m taking this [ECPs] and I don’t want to have to keep on doing this...this is not an effective method. This is not thinking ahead, and I wanted to be able to think ahead from now on, or not have to think at all.”

Women shared their reluctance to rely on ECPs because of concerns about their efficacy and effects on their body, as well as a view that using ECPs was irresponsible or “not thinking ahead”. For them, the copper IUD had the characteristics they were interested in when considering a method (non-hormonal, effective, and low maintenance) and none of the drawbacks they felt ECPs presented.

## Discussion

This study aimed to understand women’s motivations for adopting either ECPs or the copper IUD at the EC visit, in settings where provision of both EC methods was standard practice and at no cost to the patient. We find that women have varied and well-considered reasons for choosing each EC method. Women who left with ECPs and those who left with the copper IUD valued each post-coital contraceptive option, yet some viewed frequent ECP use as irresponsible, unreliable, or potentially damaging to the body. Ultimately, the key reasons women gave for choosing the copper IUD over ECPs were due to awareness about it, the opportunity to have it placed, comfort with its characteristics, and lack of negative experiences with the IUD. Whereas women who chose ECPs over the IUD described lack of awareness about the IUD as EC, aversion toward IUD side effects or the placement process, or previous negative experiences using the IUD. Most women who left with ECPs planned to adopt a reliable ongoing method, either at the current visit or in the near future, that would eliminate the need for ECPs. The reasons women planned to return for contraception at a later clinic visit were because the clinic or woman was not able to have an IUD or implant placed that day, or the woman needed more time to decide on a method.

The women who chose the copper IUD at the EC visit described their readiness for an ongoing method, as well as a desire to not rely on ECPs. Unlike those who adopted ECPs, these women had already considered the IUD before having it placed at the EC visit. For them, the EC visit served as an opportunity to move their contraceptive plan forward. Given the long-acting nature of the IUD and the need for a provider to place it, choosing an IUD over ECPs arguably takes more commitment to the method, one that is easier to bridge if women are already knowledgeable about the method and feel that its characteristics line up with their contraceptive priorities. A qualitative study of nulliparous adolescents identified “first awareness”, “initial reaction” and “information gathering” stages that IUD users progressed through before being ready for IUD adoption [28]. As we found in this study, women’s contraceptive preferences and experiences gathering information from personal sources and medical providers were key influences in the process of choosing an IUD.

For two women in our study, the IUD as EC was the only effective pregnancy prevention option, given the timing of unprotected sex and their clinic visit. These women valued the IUD’s effectiveness, including its longer window of efficacy after unprotected sex and its long-term contraceptive coverage. This option is particularly helpful to provide for women who may present later after unprotected sex or who would choose the IUD as EC because of its higher efficacy compared to ECPs.

In a patient-centered healthcare model, providers offer and fully inform patients about their options and engage in a shared-decision-making approach that respects patient preferences [29]. This approach can support patients to take ownership over and feel more satisfied with their decision, as evidenced by research on patient preferences for IUD removal, contraceptive counseling and other healthcare decision-making [30–34]. With regard to EC method choices, many women do not have access to the IUD as EC, given existing structural barriers [7–9]. Even at our study sites that offer the IUD as EC as standard practice, some women reported that they were not offered, but may have chosen, the IUD as EC. Given limited access to the IUD as EC, women are unlikely to know about this option on their own [6, 8, 9, 11]. Inclusion of the IUD as an EC method when providing EC options to women can help to ensure that women have more meaningful choices.

Patient-centered counseling can also support women’s decisions to use ECPs and frame it as a positive choice, reinforcing the method's safety and destigmatizing repeat use. Even after contraceptive counseling at their clinic visit, the women in our study shared concerns that that using ECPs was unreliable and irresponsible or that ECP use could lead to fertility loss and bodily harm, concerns that have been noted in other studies [35, 36]. While efficacy is an important and valid consideration, women should be made to feel safe and supported using ECPs, as there are no known negative effects of ECPs on long-term fertility and repeated use is safe [37]. Despite the lack of contraindications, professional organizations such as the American Academy of Pediatrics and American College of Obstetricians and Gynecologists discourage repeat use of ECPs [38, 39], potentially contributing to the stigma of ECP use described by the participants in this study.

### Strengths and limitations

We chose to recruit in clinics where provision of the IUD as EC was described as standard practice, so we could hear from women who chose the IUD as EC, an understudied group of women, and to focus on the barriers to IUD as EC uptake that are not specific to access, although some of these issues still arose. Not all women we interviewed reported the clinic offered them the IUD as an EC option, and we do not know the patient or provider-level factors affecting its availability to those patients. Even without continuous availability, the high comparative level of access to the IUD as EC at our recruitment clinics may not be transferrable to other settings. However, we could apply the findings about women’s decision-making processes to choose an EC method more broadly in settings where either EC method is offered. Our study did not distinguish between ECP types, but confirmed with eight of ten ECP participants that they received a levonorgestrel ECP. In order to quell issues of trustworthiness of the data, we adhered to standardized written data collection protocols, and analyzed data using multiple coders, assessing the full context of women’s experiences, and examining our own conceptual perspectives and biases as researchers while drawing conclusions. We acknowledge our bias for conducting this research was in part to understand how to improve the provision of the IUD as EC.

## Conclusions

This study highlights that ECPs and the IUD as a form of EC are important and acceptable EC options, each with their own features offering benefits to patients. Women’s reasons for choosing ECPs over the copper IUD, such as negative IUD experiences and discomfort with IUD side effects and placement, reinforce the need to support women’s decisions to use ECPs rather than stigmatize their use. Because of these reasons, the IUD as EC is unlikely to replace ECPs as a top EC method for most women, in spite of increased access, but the copper IUD can be an effective choice for both EC and ongoing contraception if the method aligns with women’s preferences. However, we find that even in settings where provision of both ECPs and the IUD as EC is standard practice, IUD as EC access barriers remain.

We find that the women seeking EC in this study had thoughtful and valid reasons for choosing ECPs or the IUD as EC, highlighting the importance of ensuring that contraceptive counseling is centered on patient preferences, while also providing information and choices. We also found that IUD as EC access barriers persist even in settings that offer this method as standard practice, suggesting that structural barriers to IUD provision, such as availability of trained providers and time for counseling, still need to be addressed in order to ensure women have meaningful choices. Finally, efforts by providers and practices to increase patient awareness of the IUD as EC and to destigmatize the use of ECPs can help to further support women in their EC method decision-making.

## Data Availability

All relevant qualitative data are within the paper. Original interviews cannot be provided because public availability would compromise patient confidentiality and participant privacy.

## Abbreviations

*EC*: Emergency contraception
*ECPs*: Emergency contraception pills
*IUD*: Intrauterine device
